# Laser-assisted delivery of imiquimod in Brooke-Spiegler syndrome^⋆^

**DOI:** 10.1016/j.abd.2020.12.014

**Published:** 2022-05-30

**Authors:** Aysenur Botsali, Ercan Caliskan

**Affiliations:** Department of Dermatology, Gulhane Training and Research Hospital, University of Health Sciences, Ankara, Turkey

Dear Editor,

A 34-year-old female was admitted with Brooke-Spiegler Syndrome (BSS). On dermatologic examination, confluent, infiltrated papules compatible with trichoepithelioma were detected (Fig. 1A, Fig. 2A).

**Figure 1:**
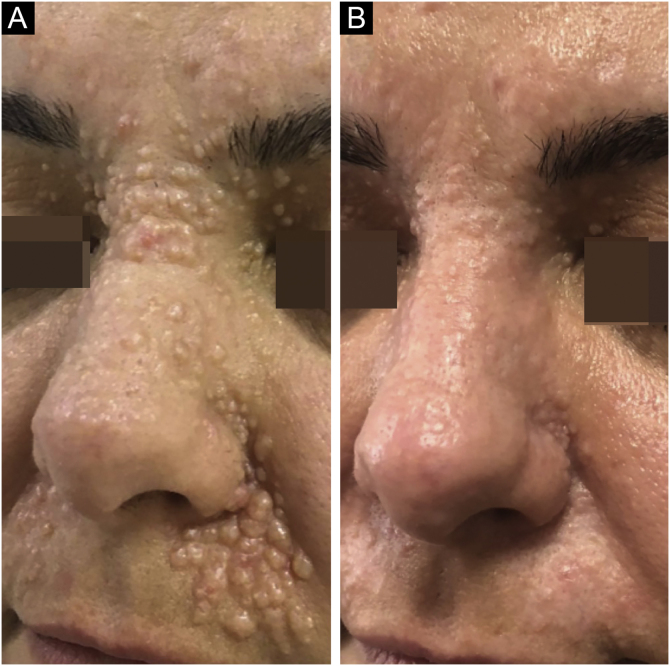
(A), On admission, multiple confluent, flesh-colored, infiltrated papules were detected on the nasal dorsum, bilateral alar grooves, and nasolabial regions. (B), At last control, the left nasolabial sulcus skin demonstrated a mild hypopigmentation. A prominent regression that was scored 80% by the patient was noted for the remaining parts receiving laser-assisted delivery of imiquimod.

**Figure 2:**
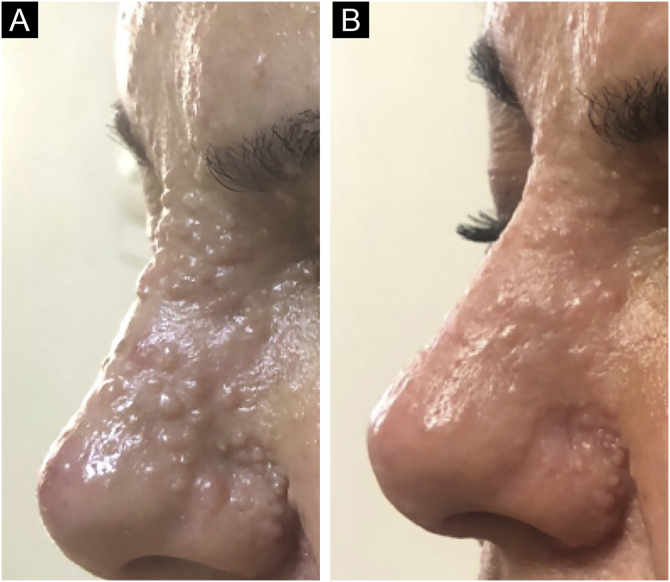
(A), A close-up image of the trichoepithelioma lesions located on the nasal dorsum. (B), The same site at the last follow-up.

Initially, the prominent lesions on the left nasolabial sulcus skin were ablated by full-field erbium: YAG laser applied at the setting of 3-mm spot-size, 6 J/cm^2^, 10 Hz with 100 μsn pulse duration (Fotona, XS, Dynamis). Due to the requirement of sedoanalgesia for the remaining lesions, she was offered fractional erbium: YAG laser and topical imiquimod combination.

The pre-determined 4-week treatment cycles were repeated for 6-months as monthly Ablative Fractional Laser (AFL) applications immediately followed by imiquimod cream applied 5 consecutive days per week for the first 2-weeks. This was followed by a treatment-free period of 2-weeks. Severe irritation occurred that subsided during treatment-free periods. On 6-months control, her self-reported score changed from 10 to 0 for the part treated with full-field resurfacing and 10 to 2 for the parts treated with a combination of AFL and imiquimod (Fig. 1). She has been under follow-up for 1.5 years without recurrence (Fig. 1B, Fig. 2B).

BSS is an autosomal dominant disorder characterized by progressive, stigmatizing benign cutaneous neoplasms localized on the facial skin and the scalp.

Surgical excision and alternative destructive modalities were reported for BSS skin neoplasms.^1^ The ideal goal of interventions is to remove lesions without scarring and dyspigmentation, still yet efficacious enough to prevent recurrences. Recently, topical sirolimus and imiquimod treatments were suggested as non-invasive approaches.^1^, ^2^, ^3^, ^4^

The first observation on the efficacy of imiquimod was reported in a case with Multiple Facial Trichoepitheliomas (MFT).^4^ Topical tretinoin was introduced in the 6^th^ month to improve the penetration of imiquimod. This combination was continued till 3^rd^ year. A retrospective study reported partial response after 32-weeks of imiquimod treatment in two BSS patients.^2^ These treatment durations are significantly longer when compared to the approved indications of imiquimod (Table 1). In a single-subject case study, the efficacy of various treatments was evaluated. Two sites received either imiquimod or the combination of AFL and imiquimod. On the 3^rd^ month, topical imiquimod wasn’t effective. The treatment result of AFL and imiquimod was also similar to AFL alone. An important difference from the combination design is that imiquimod wasn't applied immediately, instead 2 days after AFL.^1^ AFL is shown to increase imiquimod flux 46 to 127-fold through multiple passes.^5^ Immediate application of the topical agent is central to treatment success of Laser-Assisted Delivery (LAD) which may explain discrepancies in treatment results. A limitation of current observation is that AFL alone can provide similar results, which cannot be compared to the combination therapy defined for this case. However, it should be emphasized that the lack of recurrence on long-term follow-up is extremely rare for Brooke-Spiegler syndrome and the detected results are remarkable.

**Table 1:** Literature data on the topical treatment options of BSS/MFT.

Author, year	Type/Patient number	Treatment	Duration weeks (wks)	Result
Urquhart JL, 2005	MFT/1	IMI + Tretinoin	156	80% clearance
Alessi SS, 2009	BSS/2	IMI	34	Partial response
LoPiccolo MC, 2011	BSS/1	IMI	12	Patient stated disease severity (compared to baseline) 10/10
		PDT		7/10
		Er: YAG laser		6/10
		Er: YAG laser + IMI		6/10
		Er: YAG laser + PDT		5/10
Tu JH et al., 2016	MFT/2	CO2 laser + Sirolimus	52	Limited re-growth
		Sirolimus	30	Limited disease progression

MFT, Multiple Facial Trichoepithelioma; BSS, Brooke-Spiegler Syndrome; IMI, Imiquimod; PDT, Photodynamic Therapy.

As imiquimod requires extended treatment durations for trichoepitheliomas, LAD can assist in decreasing the duration. Another important consideration about this regimen is the lack of recurrence on long-term follow-up.

## Financial support

None declared.

## Authors' contributions

Aysenur Botsali: Approval of the final version of the manuscript; Critical literature review; Data collection, analysis, and interpretation; Effective participation in research orientation; Intellectual participation in propaedeutic and/or therapeutic management of studied cases; Preparation and writing of the manuscript; Study conception and planning.

Ercan Caliskan: Approval of the final version of the manuscript; Critical literature review; Effective participation in research orientation; Intellectual participation in propaedeutic and/or therapeutic management of studied cases; Manuscript critical review; Study conception and planning.

## Conflicts of interest

None declared.
